# Impact of *ABCG2* Gene Polymorphism on the Predisposition to Psoriasis

**DOI:** 10.3390/genes12101601

**Published:** 2021-10-12

**Authors:** Yu-Huei Huang, Lai-Chu See, Ya-Ching Chang, Wen-Hung Chung, Lun-Ching Chang, Shun-Fa Yang, Shih-Chi Su

**Affiliations:** 1 Institute of Medicine, Chung Shan Medical University, Taichung 402, Taiwan; huangyh@cloud.cgmh.org.tw; 2 Department of Dermatology, Chang Gung Memorial Hospital, Linkou Branch, Taoyuan 333, Taiwan; ycchang@cgmh.org.tw (Y.-C.C.); chung1@cgmh.org.tw (W.-H.C.); 3 School of Medicine, College of Medicine, Chang Gung University, Taoyuan 333, Taiwan; 4 Department of Public Health, Chang Gung University, Taoyuan 333, Taiwan; lichu@mail.cgu.edu.tw; 5 Biostatistics Core Laboratory, Molecular Medicine Research Center, Chang Gung University, Taoyuan 333, Taiwan; 6 Division of Rheumatology, Allergy and Immunology, Department of Internal Medicine, Chang Gung Memorial Hospital, Linkou Branch, Taoyuan 333, Taiwan; 7 Whole-Genome Research Core Laboratory of Human Diseases, Chang Gung Memorial Hospital, Keelung 204, Taiwan; 8 Department of Mathematical Sciences, Florida Atlantic University, Boca Raton, FL 33431, USA; changl@fau.edu; 9 Department of Medical Research, Chung Shan Medical University Hospital, Taichung 402, Taiwan

**Keywords:** psoriasis, single nucleotide polymorphisms, *ABCG2*

## Abstract

Psoriasis is a chronic inflammatory disease which is caused by the interaction between genetic and environmental factors. Evidence shows an association of psoriasis with co-morbidities including cardiovascular diseases, metabolic syndrome and hyperuricemia. Genome-wide association studies have revealed that the *ABCG2* gene encoding ATP-binding cassette G2 protein was associated with inflammation and higher serum urate concentrations. In this study, we aimed to evaluate the role of *ABCG2* gene polymorphisms on the susceptibility to psoriasis. The genotype distribution of two *ABCG2* single nucleotide polymorphisms (SNPs), rs2231142 and rs2231137, was examined in 410 psoriasis patients and 1,089 gender-matched non-psoriasis controls. We found that heterozygotes (GT) for rs2231142 was associated with a decreased risk of psoriasis (*p* = 0.001; adjusted OR = 0.532; 95% CI, 0.370–0.765) after adjusting for age, as compared with homozygotes for the major allele (GG). Subjects who carried at least one polymorphic allele (homozygote or heterozygote for the minor allele) were less susceptible to psoriasis (*p* = 0.002; adjusted OR = 0.594; 95% CI, 0.249–0.823) and bearing higher serum urate levels (*p* = 0.026) than those homozygous for the major allele. Our results indicated that the *ABCG2* gene polymorphism was associated with the risk of psoriasis.

## 1. Introduction

Psoriasis is an inflammatory and multifactorial disease, which is caused by the interaction between genetic and environmental factors [1,2]. Linkage studies have uncovered nine genomic regions, known as psoriasis susceptibility (PSORS) 1–9 which were supposed to contribute to disease susceptibility [3]. Recent advances in the genome-wide associated studies (GWAS) identified more than 40 psoriasis susceptibility loci. Proteins encoded by genes at these loci were linked to regulate the inflammatory pathways and skin barrier function involved in the development of psoriatic plaques [4]. Cytokine members of the IL-23/IL-17 family were known to be substantial in driving skin inflammation due to the success of IL-23- or IL-17-targeted biologics in treating psoriasis [5]. IL-23 expressed by dendritic cells is crucial for Th17 development and expansion. Th17-driven IL-17A and IL-22 act on keratinocytes to induce CC chemokine 20 and attract Th17 cells for further keratinocyte activation in a positive feedback manner [6]. These cytokine-signaling pathways were associated with several psoriasis susceptibility loci such as *IL23A*, *IL23R*, and *NFKBIZ* [7,8,9]. The findings of these risk alleles support a role of IL-23 and IL-17 in psoriasis pathogenesis.

The *ABCG2* (ATH-binding cassette G2) protein, a half transporter, is one member of ABC (ATP-binding cassette) transporters that export multiple compounds, including lipids, amino acids, uric acid, inorganic ions, chemotherapeutics [10], and environmental and endogenous toxins [11,12] across membranes. ABC transporters were initially identified for their roles in the onset and maintenance of multidrug resistance. Several reports have shown their contributions to the development, differentiation, and maturation of immune cells and their involvement in migration of immune effector cells [13]. The presence of these transporters on immune cells could be implicated in the onset of autoimmune diseases by active secretion of inflammatory mediators such as prostaglandins, leukotrienes, and cyclic nucleotides (cAMP, cGMP) [14,15]. Numerous studies have demonstrated that *ABCG2*, expressed on hematopoietic stem cells, monocyte-derived dendritic cells, and Langerhans cells, has played an important role in their differentiation, maturation, and migration [16,17]. In inflammatory conditions, ABC transporters influence the susceptibility to development of rheumatoid arthritis [18,19] where marked expression of *ABCG2* in the intimal lining layer and on macrophages in the synovial tissue were observed [20]. Increased expression of *ABCG2* in peripheral blood mononuclear cells was described in psoriasis as well [21]. All these findings implicate *ABCG2* as not only a drug efflux pump but a critical factor in inflammatory processes and autoimmune diseases.

Here, two commonly-studied missense polymorphisms (V12M; rs2231137 and Q141K; rs2231142) from the *ABCG2* gene were selected on the basis of their potential involvement in the susceptibility of hyperuricemia for examining their association with psoriasis [12]. These two missense variants of the *ABCG 2* gene were shown to have significant impacts on *ABCG2* characteristics, leading to reduced *ABCG2* protein expression and dysfunction. [22,23]. *ABCG2* genetic variants have been reported to be associated with reduced efficacy of drug treatments and risks of diseases, such as gout, Alzheimer’s disease, and isolated septal defects [24,25,26,27]. Therefore, the aim of this study was to test the hypothesis that *ABCG2* genetic polymorphism may potentially confer the susceptibility to psoriasis. Through assessing the genotype distribution of two *ABCG2* single nucleotide polymorphisms (SNPs), rs2231142 and rs2231137, in 410 psoriatic patients and 1089 controls, we demonstrated that *ABCG2* gene polymorphism was associated with the predisposition to psoriasis.

## 2. Materials and Methods

### 2.1. Subjects

The study was comprised of 410 patients with psoriasis and 1089 gender-matched, psoriasis-free controls with the approval by the institutional review board of Chang Gung Medical Foundation, Taiwan (No. 201503178B0). The psoriasis patients were recruited in the Chang Gung Memorial Hospital from 2017 to 2019 and the control subjects were enrolled in the Chang Gung Memorial Hospital and selected from the Integrated Blood Bank of the Chang Gung University. Patients received physical examination and laboratory blood tests including uric acid, high-density lipoprotein, low-density lipoprotein, total cholesterol, and triglycerides upon recruitment. Psoriasis severity was measured by using the psoriasis area and severity index (PASI) score. Clinical information regarding age, gender, coexistence of psoriatic arthritis, and habits of smoking and alcohol drinking was collected.

### 2.2. Genotyping of ABCG2 SNPs

Genomic DNA was extracted from the whole blood with QIAamp DNA Blood Mini Kits (Qiagen, Santa Clarita, CA, USA), as described in detail previously [28]. DNA was dissolved in Tris-EDTA buffer and then quantified by a measurement of OD260. Analysis of allelic discrimination for the two *ABCG2* SNPs (rs2231142 and rs2231137) was performed by using the TaqMan assay with an ABI StepOne Real-Time PCR System (Applied Biosystems, Foster City, CA, USA) and then assessed with SDS 3.0 software (Applied Biosystems).

### 2.3. Statistical Analysis

The differences in demographic parameters between patients with psoriasis and controls were estimated by using the Mann- Whitney U test and Fisher’s exact test. Adjusted odds ratios (AORs) and their 95% confidence intervals (CIs) for the association between genotype frequencies and the risk of psoriasis were calculated by multiple logistic regression models after controlling for other covariates. Data were analyzed with SAS 9.1 statistical software (SAS Institute Inc., Cary, NC, USA). A *p* value < 0.05 was considered significant.

## 3. Results

### 3.1. Characterization of Study Participants

In this study, 410 psoriasis patients and 1089 gender-matched non-psoriatic controls were enrolled. Demographic and clinical characteristics are presented in Table 1. In addition to age, significant differences in several metabolism-related parameters such as body mass index, weight, and the levels of high-density lipoprotein, total cholesterol, and triglycerides were observed between two study cohorts.

### 3.2. Association between ABCG2 Gene Polymorphisms and Psoriasis

To understand the possible association of *ABCG2* gene polymorphisms with the risk of psoriasis, the genotype distributions of two SNPs, rs2231142 and rs2231137, were examined (Table 2). We observed that Heterozygous participants (GT) for rs2231142 were associated with a decreased risk of psoriasis (*p* = 0.001; adjusted OR = 0.532; 95% CI, 0.370–0.765) as compared to homozygotes for the major allele (GG) after adjusting for age. Furthermore, subjects who carried at least one polymorphic allele (homozygote or heterozygote for the minor allele, GT + TT) were less susceptible to psoriasis than those homozygous for the major allele (*p* = 0.002; adjusted OR = 0.594; 95% CI, 0.249–0.823). However, no significant association between psoriasis and rs2231137 was detected. These results indicate a protective impact of *ABCG2* polymorphisms on psoriasis.

### 3.3. Interaction of ABCG2 Gene Polymorphisms with Clinical Characteristics among Patients with Psoriasis

Since a genetic predisposition to psoriasis was noted, we further analyzed the effect of *ABCG2* gene polymorphisms on clinical characteristics in patients with psoriasis (Table 3 and Table 4). A significant association of rs2231142 variants (GG vs. GT + TT) with hyperuricemia (*p* = 0.026; OR = 1.608, 95% CI: 1.057–2.447) was observed in psoriasis patients. However, such association of rs2231142 variants was not demonstrated with age of onset, family history of psoriasis, baseline PASI score, or psoriatic arthritis. 

## 4. Discussion

The present study, for the first time, investigated the role of *ABCG2* polymorphism as a possible genetic risk factor for psoriasis. We found significant differences in genotype frequencies of *ABCG2* rs2231142 between the psoriasis group and control population. Specifically, GT/TT genotypes of *ABCG2* rs2231142 were associated with a reduced risk of psoriasis and were more prone to develop hyperuricemia in psoriasis patients. The correlation between GT/TT genotypes of the rs2231142 polymorphism and increased serum urate levels found in our cohort was consistent with previous reports in the Japanese and Han Chinese populations [29,30].

The link between psoriasis and *ABCG2* gene polymorphisms implies that the polymorphic alleles may possess a protective effect from developing this cutaneous disease. Intriguingly, similar findings concerning the association of psoriasis with the *IL12B* and *IL23R* gene polymorphisms were documented in previous studies [31,32]. The polymorphic allele of *ABCG2* rs2231142 is a missense variant that leads to a glutamine-to-lysine amino acid substitution (Q141K) in the exon 5 as a consequence of contributing to lower *ABCG2* protein expression [22,33]. The role of *ABCG2* in inflammatory diseases has been described in rheumatoid arthritis and psoriasis [19,20,21]. The phenomenon that functionality of *ABCG2* was correlated with the disease activity in patients with recently diagnosed rheumatoid arthritis could be due to an inherent feature of lymphocytes [19]. The observation of marked *ABCG2* expression in peripheral mononuclear cells from psoriasis seems to be consistent with our genetic findings since the predominant genotype (GG), known to be associated with higher transcription activities, was found to be more common in the psoriasis population then the control group. However, these results are contradictory to previous studies indicating a suppressive effect of *ABCG2* on inflammatory signaling pathways [2]. To solve this contradiction, further in-depth investigation on the role of *ABCG2* in psoriasis pathogenesis is warranted. Moreover, another *ABCG2* SNP rs1448784 was located within the 3′-untranslated region and found to confer great susceptibility to gout [34]; this could be taken into consideration in future studies, in addition to the two most commonly studied missense SNPs, rs2231137 and rs2231142.

High levels of serum uric acid are frequently observed in patients with psoriasis. However, the actual causal relationship between psoriasis and hyperuricemia remains unknown. As psoriasis and hyperuricemia are affected by a number of shared and separated genetic factors, a significantly higher level of uric acid was detected in psoriasis patients from the west but not from middle Asia and India in comparison with controls [35,36], indicating an ethnicity-specific correlation between psoriasis and hyperuricemia. In psoriasis, uric acid is considered as a byproduct of rapid skin cell turnover and systemic inflammation. Our observation that patients who carried at least one polymorphic allele (presumably leading to lower *ABCG2* protein expression) of rs2231142 showed higher serum urate levels is in concert with the proposed function of *ABCG2* as a high-capacity urate exporter. It is worth noting that, while no significant difference in serum urate levels was seen between our case and the control group, our genotyping results revealed a protective effect of the *ABCG2* genetic polymorphism on psoriasis.

Our data revealed an influence of *ABCG2* gene variations on the predisposition to psoriasis; however, there are several limitations in this study. First, the findings reported in this study may be unable to be extended to other ethnic groups unless replication cohorts are assessed. Second, the control group in this hospital-based study was recruited from individuals that may have psoriasis-like skin conditions which we could not exclude entirely. Another issue is that since the difference in age between the two study groups was significant and considerable, we corrected the associations for age but did not correct for other parameters. Additionally, more functional experiments are needed to determine the role of *ABCG2* in the pathogenesis of psoriasis.

## 5. Conclusions

In conclusion, the present study revealed the novel finding that *ABCG2* rs2231142 polymorphism was associated with psoriasis, indicating a link of altered *ABCG2* expression or function to psoriasis pathogenesis.

## Figures and Tables

**Table 1:** Distributions of demographic characteristics of 1089 control and 410 patients with psoriasis.

Variable	Control (*n* = 1089)	Patients (*n* = 410)	*p* Value
Age (years)	Mean ± S.D.	Mean ± S.D.	
	54.22 ± 11.09	41.40 ± 12.55	*p* < 0.001
Gender			
Male	765 (70.2%)	291 (71.0%)	*p* = 0.783
Female	324 (29.8%)	119 (29.0%)	
Height (cm)	163.35 ± 8.16	167.26 ± 8.03	*p* < 0.001
Weight (kg)	68.99 ± 12.23	74.34 ± 15.69	*p* < 0.001
Body mass index (BMI)	25.79 ± 3.84	26.50 ± 4.93	*p* = 0.004
Uric acid (mg/dL)	6.22 ± 1.42	6.34 ± 1.65	*p* = 0.161
high-density lipoprotein (HDL, mg/dL)	56.01 ± 14.82	47.42 ± 11.58	*p* < 0.001
low-density lipoprotein (LDL, mg/dL)	117.32 ± 27.69	116.59 ± 35.99	*p* = 0.825
Total cholesterol (mg/dL)	202.16 ± 37.30	192.43 ± 63.16	*p* < 0.001
Triglycerides (mg/dL)	151.86 ± 125.22	136.82 ± 145.77	*p* = 0.048
PASI score		11.54 ± 9.86	
Onset (age, on skin)		27.84 ± 12.89	
Arthritis pain			
No		275 (67.1%)	
Yes		135 (32.9%)	

**Table 2:** Distribution of *ABCG2* genotype frequencies in 1089 controls and 410 psoriasis patients.

Variable	Controls (*n* = 1089) *n* (%)	Patients (*n* = 410) *n* (%)	OR (95% CI)	AOR (95% CI)
*ABCG2* rs2231142				
GG	523 (48.0%)	234 (57.1%)	1.00	1.00
GT	445 (40.9%)	137 (33.4%)	0.688 (0.538–0.880) *p* = 0.030	0.532 (0.370–0.765) *p* = 0.001
TT	121 (11.1%)	39 (9.5%)	0.720 (0.487–1.067)	0.812 (0.485–1.358)
GT + TT	566 (52.0%)	176 (42.9%)	0.695 (0.553–0.874) *p* = 0.002	0.594 (0.429–0.823) *p* = 0.002
*ABCG2* rs2231137				
CC	486 (44.6%)	180 (43.9%)	1.00	1.00
CT	476 (43.7%)	180 (43.9%)	1.021 (0.801–1.301)	0.928 (0.656–1.313)
TT	127 (11.7%)	50 (12.2%)	1.063 (0.735–1.538)	1.124 (0.681–1.856)
CT + TT	603 (55.4%)	230 (56.1%)	1.030 (0.819–1.295)	0.943 (0.665–1.337)

The odds ratio (OR) with 95% confidence intervals (CIs) were estimated by logistic regression models. The adjusted OR (AOR) with their 95% CIs was estimated by multiple logistic regression models after controlling for age.

**Table 3:** Distribution of *ABCG2* rs2231142 genotype frequencies and the clinical status among 410 patients with psoriasis.

	*ABCG2* (rs2231142)			
Variable	GG (%) (*n* = 234)	GT + TT (%) (*n* = 176)	OR (95% CI)	*p* Value
Uric acid ^#^				
<7 mg/dL	170 (72.6%)	109 (62.3%)	1.00	
≥7 mg/dL	64 (27.4%)	66 (37.7%)	1.608 (1.057–2.447)	*p* = 0.026
Family History				
None	159 (67.9%)	131 (74.4%)	1.00	
Parent/Children	37 (15.8%)	24 (13.6%)	0.787 (0.448–1.383)	*p* = 0.405
Others	38 (16.2%)	21 (11.9%)	0.671 (0.375–1.199)	*p* = 0.176
PASI ^#^				
<10	128 (54.9%)	99 (56.3%)	1.00	
≥10	105 (45.1%)	77 (43.7%)	0.948 (0.639–1.406)	*p* = 0.791
Onset (age, on skin)				
<40	198 (84.6%)	145 (82.4%)	1.00	
≥40	36 (15.4%)	31 (17.6%)	1.176 (0.695–1.989)	*p* = 0.546
Arthritis pain				
No	150 (64.1%)	125 (71.0%)	1.00	
Yes	84 (35.9%)	51 (29.0%)	0.729 (0.478–1.110)	*p* = 0.140

^#^*n* = 409.

**Table 4:** Distribution of *ABCG2* rs2231137 genotype frequencies and the clinical status among 410 patients with psoriasis.

	*ABCG2* (rs2231137)			
Variable	CC (%) (*n* = 180)	CT + TT (%) (*n =* 180)	OR (95% CI)	*p* Value
Uric acid ^#^				
<7 mg/dL	114 (63.7%)	124 (68.9%)	1.00	
≥7 mg/dL	65 (36.3%)	56 (31.1%)	0.792 (0.511–1.228)	*p* = 0.297
Family History				
None	132 (73.3%)	130 (72.2%)	1.00	
Parent/Children	21 (11.7%)	23 (12.8%)	1.112 (0.587–2.107)	*p* = 0.745
Others	27 (15.0%)	27 (15.0%)	1.015 (0.565–1.824)	*p* = 0.959
PASI ^#^				
<10	97 (53.9%)	99 (55.3%)	1.00	
≥10	83 (46.1%)	80 (44.7%)	0.944 (0.623–1.431)	*p* = 0.787
Onset (age, on skin)				
<40	151 (83.9%)	153 (85.0%)	1.00	
≥40	29 (16.1%)	27 (15.0%)	0.919 (0.519–1.625)	*p* = 0.771
Arthritis pain				
No	127 (70.6%)	114 (63.3%)	1.00	
Yes	53 (29.4%)	66 (36.7%)	1.387 (0.892–2.157)	*p* = 0.145

^#^*n* = 409.

## Data Availability

The data presented in this study are available on request from the corresponding author.
